# Combined resection of the hepatic artery without reconstruction in pancreaticoduodenectomy: a case report of pancreatic cancer with an aberrant hepatic artery

**DOI:** 10.1186/s40792-020-00997-5

**Published:** 2020-09-29

**Authors:** Tadao Kuribara, Tatsuo Ichikawa, Kiyoshi Osa, Takeshi Inoue, Satoshi Ono, Kozo Asanuma, Shiori Kaneko, Takayuki Sano, Itaru Shigeyoshi, Kouta Matsubara, Naoko Irie, Akira Iai, Tetsuya Shinobi, Hideki Ishizu, Katsuhiro Miura

**Affiliations:** 1 Department of Surgery, Saitama Cooperative Hospital, 1317 Kizoro, Kawaguchi-shi, Saitama, 333-0831 Japan; 2 Department of Internal Medicine, Saitama Cooperative Hospital, 1317 Kizoro, Kawaguchi-shi, Saitama, Japan; 3 Department of Pathology, Saitama Cooperative Hospital, 1317 Kizoro, Kawaguchi-shi, Saitama, Japan; 4 Tumor Center, Nihon University Itabashi Hospital, 30-1, Oyaguchikamicho, Itabashi-ku, Tokyo, Japan; 5 Department of Hematology and Rheumatology, Nihon University School of Medicine, 30-1, Oyaguchikamicho, Itabashi-ku, Tokyo, Japan

**Keywords:** Pancreatic cancer, Pancreaticoduodenectomy, Conversion surgery, Hepatic arterial resection, Aberrant hepatic artery, Embolization, Chemotherapy

## Abstract

**Background:**

Pancreaticoduodenectomy (PD) is rarely performed for pancreatic cancer with hepatic arterial invasion owing to its poor prognosis and high surgical risks. Although there has been a recent increase in the reports of PD combined with hepatic arterial resection due to improvements in disease prognosis and operative safety, PD with major arterial resection and reconstruction is still considered a challenging treatment.

**Case presentation:**

A 61-year-old man with back pain was diagnosed with pancreatic head and body cancer. Although distant metastasis was not confirmed, the tumor had extensively invaded the hepatic artery; therefore, we diagnosed the patient with locally advanced unresectable pancreatic cancer. After gemcitabine plus nab-paclitaxel (GnP) therapy, the tumor considerably decreased in size from 35 to 20 mm. Magnetic resonance imaging revealed a gap between the tumor and the hepatic artery. Tumor marker levels returned to their normal range, and we decided to perform conversion surgery. In this case, an artery of liver segment 2 (A2) had branched from the left gastric artery; therefore, we decided to preserve A2 and perform PD combined with hepatic arterial resection without reconstruction. After four cycles of GnP therapy, we performed hepatic arterial embolization to prevent postoperative ischemic complications prior to surgery. Immediately after embolization, collateral arterial blood flow to the liver was observed. Operation was performed 19 days after embolization. Although there was a temporary increase in liver enzyme levels and an ischemic region was found near the surface of segment 8 of the liver after surgery, no liver abscess developed. The postoperative course was uneventful, and S-1 was administered for a year as adjuvant chemotherapy. The patient is currently alive without any ischemic liver events and cholangitis and has not experienced recurrence in the past 4 years since the surgery.

**Conclusions:**

In PD for pancreatic cancer with hepatic arterial invasion, if a part of the hepatic artery is aberrant and can be preserved, combined resection of the common and proper hepatic artery without reconstruction might be feasible for both curability and safety.

## Background

Surgical resection is essential for the long-term survival of patients with pancreatic cancer. However, in several cases, the tumor is unresectable at the time of diagnosis. In recent years, the progress in chemotherapy has been remarkable, and many reports of conversion surgery have been published [1–3]. It has been reported that the prognosis of patients with locally advanced pancreatic cancer (LAPC) who undergo conversion surgery is not inferior to that of patients with initially resectable cancer [1, 4]. In the Japanese 2019 Guidelines for the Treatment of Pancreatic Cancer [5], conversion surgery for LAPC has been proposed as a treatment option.

In conversion surgery, preserving blood vessels that have been invaded before treatment is a risk of vascular injury during surgery and can leave cancer cells at the resection margins. Therefore, combined vessel resection may be performed. Although arterial reconstruction may be performed to maintain organ blood flow after arterial en bloc resection, it is a difficult procedure compared with portal vein (PV) reconstruction, resulting in serious complications, such as postoperative bleeding and ischemia due to vessel obstruction. On the other hand, it has been reported that combined resection of the hepatic artery can be safely performed without arterial reconstruction in pancreaticoduodenectomy (PD) when combined with preoperative arterial embolization or when there is collateral blood flow [6–10].

Here, we report a case of LAPC with an aberrant artery of liver segment 2 (A2) that was treated with conversion surgery combined with hepatic arterial resection without reconstruction.

## Case presentation

**Fig. 1:**
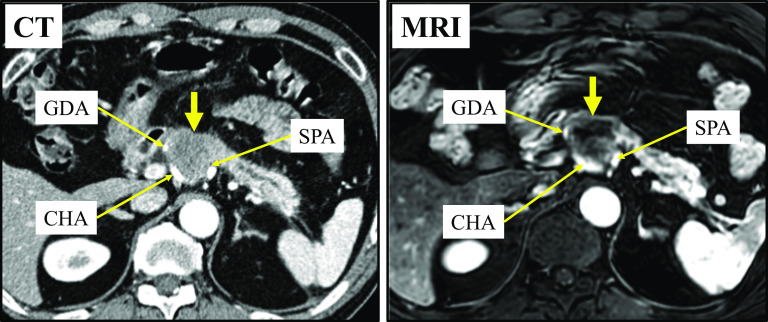
Contrast-enhanced computed tomography (CT) and magnetic resonance imaging (MRI) showing a 35-mm hypovascular tumor (yellow arrow). The tumor is in contact with the GDA, CHA, and SPA. *CHA* common hepatic artery, *GDA* gastroduodenal artery, *SPA* splenic artery

A 61-year-old man with no significant medical history presented to our hospital with back pain that started a week prior to his visit. Physiological and laboratory examinations were unremarkable except for elevated serum carbohydrate antigen 19-9 (CA19-9) and s-pancreas-1 antigen (SPan-1) levels (CA19-9, 141 U/mL; SPan-1, 110.8 U/mL). Contrast-enhanced abdominal computed tomography (CT) and gadoxetic acid-enhanced magnetic resonance imaging (MRI) revealed the presence of a 35-mm hypovascular tumor in the head and body of the pancreas, with dilatation of the main pancreatic duct and atrophy of the pancreatic tail. The tumor was observed to be in contact with the entire length of the common hepatic artery (CHA), the proximal portion of the proper hepatic artery (PHA), the gastroduodenal artery (GDA), and the root of the splenic artery (SPA) (Fig. 1). Distant metastasis was not observed. Based on these findings, the patient was diagnosed with unresectable LAPC.

**Fig. 2:**
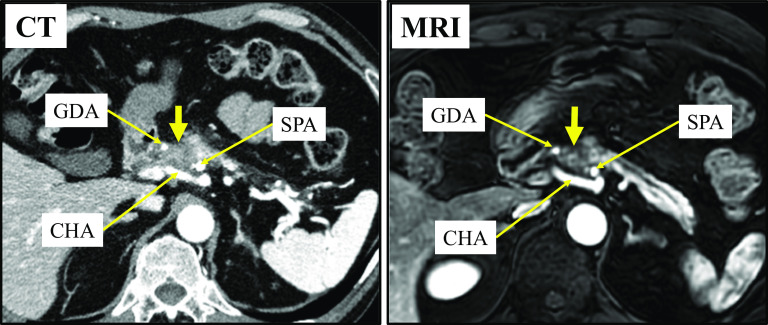
Contrast-enhanced computed tomography (CT) and magnetic resonance imaging (MRI) after two cycles of gemcitabine plus nab-paclitaxel therapy showing substantial shrinkage of the tumor (yellow arrow). Although the tumor is still in contact with the GDA, CHA, and SPA on CT, MRI shows a gap between the tumor and arteries. *CHA* common hepatic artery, *GDA* gastroduodenal artery, *SPA* splenic artery

We performed systemic chemotherapy consisting of gemcitabine plus nab-paclitaxel (GnP) (gemcitabine 1000 mg/m^2^ and nab-paclitaxel 125 mg/m^2^ on days 1, 8, and 15 every 4 weeks). After two treatment cycles, the tumor decreased in size from 35 to 20 mm. While CT revealed that the tumor was still in contact with the CHA, PHA, and SPA, contrast-enhanced MRI revealed a gap between the tumor and these arteries (Fig. 2). No distant metastasis was observed. The CA19-9 and SPan-1 levels decreased and then returned to their normal range. Based on these findings, we decided to perform conversion surgery. Next, the patient underwent another two cycles (a total of four cycles) of GnP therapy prior to surgery.

**Fig. 3:**
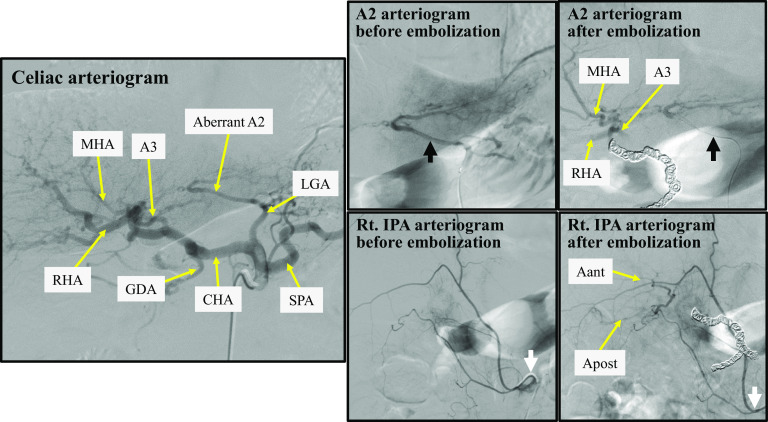
Preoperative hepatic arterial embolization. Immediately after embolization, collateral arterial flow fed by the aberrant A2 (black arrow) and the Rt. IPA (white arrow) to the whole liver is observed. Aant, artery of the right anterior section of the liver; Apost, artery of the right posterior section of the liver; A2, artery of liver segment 2; A3, artery of liver segment 3; *CHA* common hepatic artery, *GDA* gastroduodenal artery, *LGA* left gastric artery, *MHA* middle hepatic artery, *RHA* right hepatic artery, *SPA* splenic artery, *Rt. IPA* right inferior phrenic artery

The CHA was invaded by the tumor over its entire length before chemotherapy; therefore, combined resection of the hepatic artery was considered more desirable for curability and safety. Furthermore, the patient had an aberrant A2 that had bifurcated from the left gastric artery. Therefore, we planned PD with hepatic arterial resection without reconstruction. We opted to perform preoperative hepatic arterial embolization to prevent postsurgical ischemic complications. Angiography after CHA and PHA embolization immediately confirmed collateral arterial flow to the whole liver fed by the aberrant A2 and right inferior phrenic artery (Rt. IPA) (Fig. 3). Moreover, dynamic CT conducted after embolization confirmed uniform intrahepatic arterial blood flow to the whole liver.

**Fig. 4:**
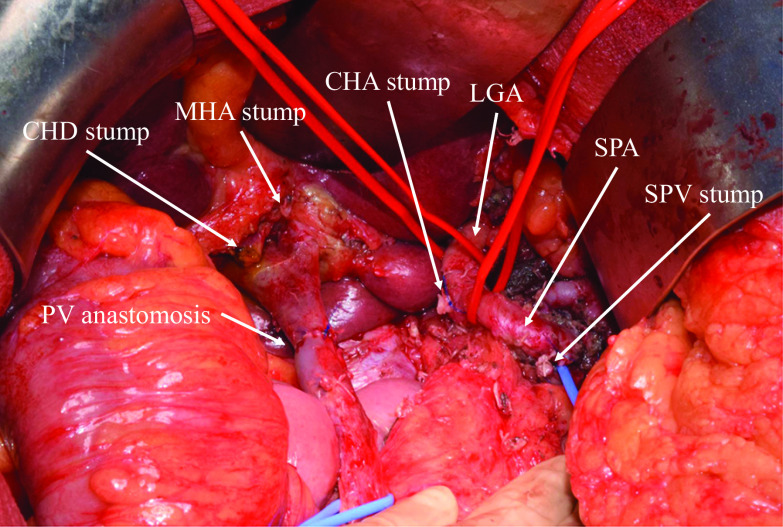
Intraoperative image obtained after tumor resection and portal vein reconstruction. *CHA* common hepatic artery, *CHD* common hepatic duct, *LGA* left gastric artery, *MHA* middle hepatic artery, *SPA* splenic artery, *PV* portal vein, *SPV* splenic vein

Nineteen days after embolization, we performed PD, PV resection with reconstruction, hepatic arterial resection without reconstruction (to preserve the aberrant A2), and D2 lymphadenectomy. The tissues around the CHA and the root of the SPA that were invaded before chemotherapy became extremely stiff; however, those around the embolized PHA were not very stiff. Therefore, the CHA was transected at its root. The distal part of the hepatic artery was transected at the root of the right, middle, and left hepatic arteries. The bile duct was transected below the bifurcation. The PV, superior mesenteric vein (SMV), and splenic vein were transected (4 cm in length), and end-to-end anastomosis of the PV and SMV was conducted with mobilization of the right colon and ileum (Fig. 4). At the end of the surgery, intraoperative Doppler ultrasonography revealed good arterial and portal flow to the whole liver. The total operation time was 947 min, and the total blood loss was 2972 mL.

**Fig. 5:**
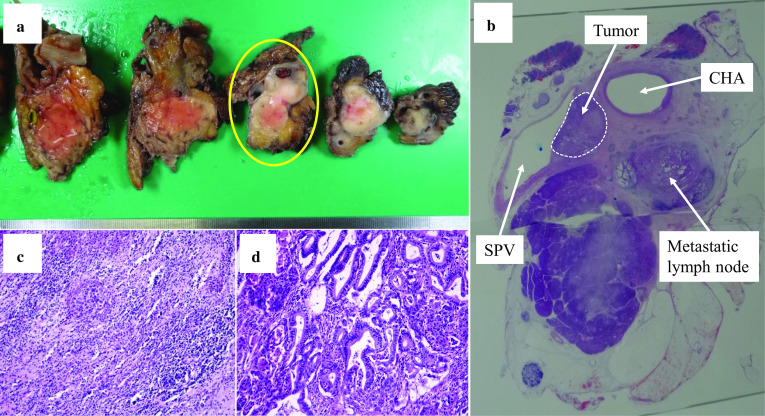
Pathological findings. **a** Macroscopic findings of the resected specimen. **b** Image of the yellow circled section in **a** as visualized with a loupe. **c** Area of the tumor in which the chemotherapy effects can be observed (H&E, × 100). **d** Area of the tumor in which viable tumor cells can be observed (H&E, × 100). *CHA* common hepatic artery, *SPV* splenic vein

Histopathological examination revealed an invasive ductal carcinoma, tub2 > por, Pbh, TS2 (40 mm), infiltrative type, ypT3, int, INFb, ly1, v1, ne0, mpd0, ypCH0, ypDU0, ypS0, ypRP0, ypPV1 (PVsp), ypA0, ypPL0, ypOO0, ypPCM0, ypBCM0, ypDPM0, ypN1a (3/11), ypM0, ypStage IIB according to the Japan Pancreatic Society classification, 7^th^ edition. According to the UICC TNM classification (8^th^ edition), the tumor was defined as T2N1M0, stage IIB. No carcinoma cells were detected in the surgical margin (R0). Histologically, this tumor was categorized as Evans grade I (Fig. 5).

Although liver enzymes levels were slightly increased and CT performed on the 7^th^ day after surgery revealed the presence of a small ischemic area in liver segment 8, no liver abscess developed. The postoperative course was uneventful, and the patient was discharged 17 days after the surgery. He received S-1 (60 mg, orally administered twice a day for 28 days followed by a 14 day rest, every 6 weeks [one cycle]) as adjuvant chemotherapy for a year and is currently alive with no evidence of recurrence and without any ischemic liver events and cholangitis within the last 4 years since his surgery.

## Discussion

Although the prognosis of patients who are initially diagnosed with unresectable LAPC remains to be poor, there are some cases of LAPC that have been successfully treated with conversion surgery after systemic chemotherapy or chemoradiotherapy, resulting in long-term survival [11–13].

In this case, the patient’s tumor was considered unresectable because it had invaded the CHA, the proximal portion of the PHA, the GDA, and the root of the SPA, as revealed by initial examination. We performed systemic chemotherapy consisting of GnP. After two cycles of GnP therapy, the tumor considerably decreased in size, tumor marker levels decreased to the normal range, and no distant metastases were observed. Therefore, we planned for conversion surgery. The progression-free survival of patients treated with GnP has been reported to be approximately 6 months [14, 15]. We performed conversion surgery after a total of four cycles (4 months) of chemotherapy. In our case, the arteries appeared detached from the tumor on MRI, unlike on CT scan, after systemic chemotherapy. The resected specimen revealed tissue degeneration at the tumor periphery. The changes observed upon MRI may reflect these pathological findings. In conversion surgery for LAPC, preservation of blood vessels that have invaded before preoperative treatment is a high-risk procedure that may result in vascular injury. Major vascular injury can cause massive bleeding and lead to serious complications as well as perioperative death. In addition, preservation of blood vessels can increase the likelihood that cancer cells will remain at the surgical margin. Considering these points and in terms of safety and curability, it may be a better choice to perform combined vascular resection. If a major vessel is resected, whether the vessel requires reconstruction should also be considered. Resection and reconstruction of the PV is considered safe if performed by an experienced surgeon [16, 17]. On the contrary, arterial reconstruction is a challenging procedure, except for a cardiovascular or plastic surgeon, with risks of serious complications, such as postoperative bleeding and organ ischemia due to vessel occlusion [4, 7].

The liver receives arterial blood flow not only from the hepatic artery but also from the collateral arteries of the surrounding structures. Intrahepatic arterial blood flow communicates via the interlobar hepatic artery at the hepatic hilus and the intrahepatic translobar artery [18–22]. The hilar bile duct receives arterial blood supply from the tiny branches at the hepatic hilus [23]. Previous studies have shown that if the hepatic artery is occluded, collateral blood flow rapidly develops and compensates for the blood supply to the liver and bile duct [21, 24]. Generally, PD does not require mobilization of the liver, large dissection of the lesser omentum, transection of the bile duct above the bifurcation, and thorough lymphadenectomy at the hilar portion. Therefore, the collateral arterial pathways to the liver and hilar bile duct are usually maintained in PD. Some studies have reported that resection of the replaced right hepatic artery in PD is commonly well tolerated without reconstruction or preoperative embolization [10, 25]. Furthermore, even in patients with only an aberrant sectional artery or those without an aberrant hepatic artery, PD combined with hepatic arterial resection without reconstruction after hepatic arterial embolization has been successfully performed [6–9]. Miyazaki et al. reported 21 cases of PD combined with hepatic arterial resection. In their report, 12 patients underwent preoperative hepatic arterial embolization and were managed without hepatic arterial reconstruction. However, nine patients with severe encasement or aberrant hepatic arteries did not undergo preoperative embolization. Among these patients, eight were managed without hepatic arterial reconstruction. Therefore, they concluded that the strategies of combined arterial resection using preoperative arterial embolization might simplify the surgical procedures and reduce the various risks associated with the surgical techniques of vascular reconstruction [7]. Our patient presented with aberrant A2; therefore, a simple resection might be tolerated. However, preoperative arterial embolization was performed to prevent ischemic complications after surgery. Because embolization may also result in complications, we need to consider which procedure is safer (reconstruction vs embolization) based on each institution or case. In our case, some physicians were familiar with arterial embolization; however, there was no cardiovascular or plastic surgeon. Therefore, we opted to perform preoperative embolization.

In patients with an aberrant hepatic artery, if the blood flow from the aberrant hepatic artery and hepatic arterial collateral circulation can be preserved, the risk of ischemic complications after PD with hepatic arterial resection can be reduced. According to previous reports, the prevalence of an abnormality in which a part or whole of the left hepatic artery (LHA) branches from the left gastric artery is approximately 12%–13% [26, 27]. Aberrant LHA has been considered to be less susceptible to cancer invasion and accidental injury during surgery because of its location. Multi-detector row CT scan can detect even tiny blood vessels; therefore, it is essential prior to surgery.

In our case, angiography confirmed good arterial blood flow to the whole liver from the aberrant A2 and the Rt. IPA immediately after hepatic arterial embolization. The postoperative course was uneventful, with only a small infarct observed in the liver. Although interruption of hepatic arterial flow is commonly well tolerated, as in our patient, this is not always the case. Therefore, patients should receive treatment while ensuring safety. In a previous case, liver necrosis occurred after cutting off a segmental artery of the liver [28]. Although this procedure was not performed in our case, confirming collateral blood flow via balloon occlusion of the hepatic artery before embolization may prevent ischemic complications.

## Conclusions

In PD for pancreatic head and body cancer with hepatic arterial invasion, if a part of the hepatic artery is aberrant and can be preserved, combined resection of the CHA and PHA without reconstruction after preoperative embolization might be feasible for both curability and safety.

## Data Availability

Data sharing is not applicable to this article as no datasets were generated or analyzed during the current study.

## Abbreviations

Aant: Artery of the right anterior section of the liver
Apost: Artery of the right posterior section of the liver
A2: Artery of liver segment 2
A3: Artery of liver segment 3
CA19-9: Carbohydrate antigen 19-9
CHA: Common hepatic artery
CT: Computed tomography
GDA: Gastroduodenal artery
GnP: Gemcitabine plus nab-paclitaxel
LAPC: Locally advanced pancreatic cancer
LGA: Left gastric artery
LHA: Left hepatic artery
MHA: Middle hepatic artery
MRI: Magnetic resonance imaging
PD: Pancreaticoduodenectomy
PHA: Proper hepatic artery
PV: Portal vein
RHA: Right hepatic artery
Rt. IPA: Right inferior phrenic artery
SMV: Superior mesenteric vein
SPA: Splenic artery
SPan-1: S-pancreas-1 antigen
